# Intrahospital Transport of Critically Ill Patients with Subarachnoid Hemorrhage—Frequency, Timing, Complications, and Clinical Consequences

**DOI:** 10.3390/jcm12247666

**Published:** 2023-12-13

**Authors:** Moritz L. Schmidbauer, Tim L. T. Wiegand, Linus Keidel, Julia Zibold, Konstantinos Dimitriadis

**Affiliations:** 1 Department of Neurology, LMU University Hospital, LMU Munich, Marchioninistrasse 15, 81377 Munich, Germany; 2 Child Brain Research and Imaging in Neuroscience (cBRAIN), Department of Child and Adolescent Psychiatry, Psychosomatics, and Psychotherapy, University Hospital, Ludwig-Maximilians-Universität, 80336 Munich, Germany

**Keywords:** SAH, intrahospital transport, complications, ICP

## Abstract

Background: Patients with subarachnoid hemorrhage (SAH) often necessitate intra-hospital transport (IHT) during intensive care treatment. These transfers to facilities outside of the neurointensive care unit (NICU) pose challenges due to the inherent instability of the hemodynamic, respiratory, and neurological parameters that are typical in these patients. Methods: In this retrospective, single-center cohort study, a total of 108 IHTs were analyzed for demographics, transport rationale, clinical outcomes, and pre/post-IHT monitoring parameters. After establishing clinical thresholds, the frequency of complications was calculated, and predictors of thresholds violations were determined. Results: The mean age was 55.7 (+/−15.3) years, with 68.0% showing severe SAH (World Federation of Neurosurgical Societies Scale 5). IHTs with an emergency indication made up 30.8% of all transports. Direct therapeutic consequences from IHT were observed in 38.5%. On average, the first IHT occurred 1.5 (+/−2.0) days post-admission and patients were transported 4.3 (+/−1.8) times during their stay in the NICU. Significant parameter changes from pre- to post-IHT included mean arterial pressure, systolic blood pressure, oxygen saturation, blood glucose levels, temperature, dosages of propofol and ketamine, tidal volume, inspired oxygen concentration, Horovitz index, glucose, pH, intracranial pressure, and cerebral perfusion pressure. Relevant hemodynamic thresholds were violated in 31.5% of cases, while respiratory complications occurred in 63.9%, and neurological complications in 20.4%. For hemodynamic complications, a low heart rate with a threshold of 61/min (OR 0.96, 95% CI 0.93–0.99, *p* = 0.0165) and low doses of midazolam with a threshold of 17.5 mg/h (OR 0.97, 95% CI 0.95–1.00, *p* = 0.0232) significantly predicted adverse events. However, the model did not identify significant predictors for respiratory and neurological outcomes. Conclusions: Conclusively, IHTs in SAH patients are associated with relevant changes in hemodynamic, respiratory, and neurological monitoring parameters, with direct therapeutic consequences in 4/10 IHTs. These findings underscore the importance of further studies on the clinical impact of IHTs.

## 1. Introduction

Patients with subarachnoid hemorrhage (SAH) frequently face complications such as re-bleeding, hydrocephalus, and vasospasm early in their treatment course on the neurointensive care unit (NICU) [1]. Addressing these complications often leads to intrahospital transport (IHT) to specialized diagnostic or therapeutic units [2]. While the risk of potentially harmful changes in hemodynamics or respiratory function associated with IHTs has been acknowledged in the wider intensive care unit (ICU) population, data for the neurocritical care population, and SAH in particular, remain sparse [3]. Initial studies hint at an increased likelihood of secondary brain damage in these patients, possibly due to changes in intracranial pressure (ICP) and cerebral hemodynamics [2,3,4,5,6]. Moreover, as the substantial share of IHTs is solely conducted to perform diagnostic procedures, such as magnet resonance imaging (MRI) or computed tomography (CT), and only a small proportion will potentially have therapeutic implications, balancing clinical benefit and risk seems particularly relevant in this cohort. Yet, there remains a notable gap in the literature focusing exclusively on the SAH cohort and the distinct challenges they encounter during IHTs.

Given the potential excess risk of IHT-related complications due to the inherent hemodynamic, metabolic, respiratory, and neurological vulnerabilities on one side, and the uncertainty regarding the therapeutic implications and clinical benefits on the other side, our study intends to explore the frequency, timing, complications, and therapeutic implications of IHTs in SAH.

## 2. Materials and Methods

### 2.1. Study Design and Participants

**Figure 1:**
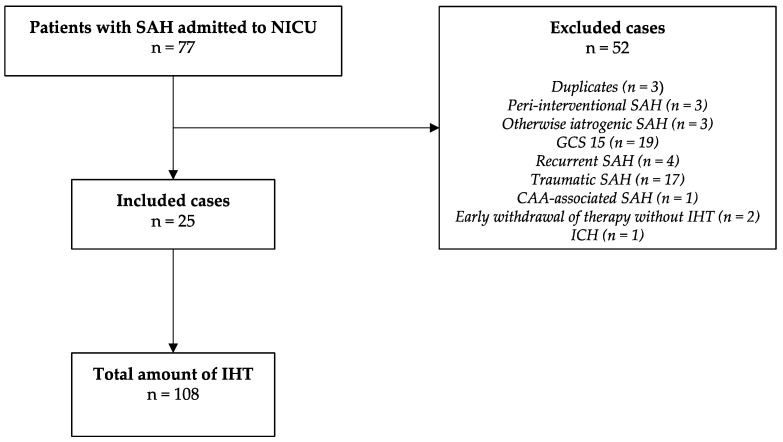
Study selection diagram. SAH—subarachnoid hemorrhage; NICU—neurointensive care unit; IHT—intrahospital transports; GCS—Glasgow Coma Scale; CAA—cerebral amlyoid angiopathy; ICH—intracerebral hemorrhage.

This study was a retrospective cohort study conducted at a tertiary center (LMU Munich) between January 2016 and April 2019. Out of 78 patients screened, 25 met our inclusion criteria (Figure 1). As patients had multiple IHTs during their stay in the NICU, data on a total of 108 IHTs were collected. Inclusion was based on admission to neurological ICU, age ≥ 18 years, primary diagnosis of non-traumatic SAH, and IHTs occurring within 14 days of admission. Patients presenting with a Glasgow Coma Scale (GCS) score of 15 were excluded. The study was conducted in accordance with the Declaration of Helsinki, and approved by the Ethics Committee of LMU Munich (protocol code 19-497, date of approval 30 July 2019).

### 2.2. Setting

All IHTs were accompanied by a physician. Monitoring was established throughout the entire process. Patient-specific targets for vital parameters were communicated to the physician conducting the IHT by the treating NICU staff. IHT physicians were equipped with emergency equipment to handle potential critical events regarding airways, breathing, circulation.

### 2.3. Data Collection

Relevant data were systematically extracted from the electronic health records (EHRs). The parameters included demographics, transport characteristics such as timing, reasons for transport, and therapeutic consequences, as well as pre/post-IHT monitoring variables. These variables consisted of GCS, mean arterial pressure (MAP), systolic blood pressure (SBP), heart rate, oxygen saturation (sO_2_), pH, carbon dioxide (CO_2_) levels, lactate (Lac), glucose (Glu), Horovitz index, temperature (T), intracranial pressure (ICP), cerebral perfusion pressure (CPP), dosage of catecholamines and sedatives, fraction of inspired oxygen (FiO_2_), tidal volume, positive end-expiratory pressure (PEEP), necessity of new central line or external ventricular drain (EVD) placements. For MAP and SBP, mean and standard deviation (SD) were calculated for 5 measurements (interval of measurement 20 min) immediately before and after IHT, respectively. For all other variables, values directly before and after IHT were obtained. Variability measures for MAP and SBP are represented by the respective SD. Data from during the IHTs were not available retrospectively. Reasons for transports were classified based on the clinical indication documented for the diagnostic or therapeutic procedure in the EHR and subsequently assigned to one of the pre-defined categories (clinical emergency, scheduled transport after intervention, post-clamping CT, other routine IHT). The most frequent indication for other routine IHTs was scheduled cranial imaging to screen for early intracranial complications (e.g., infarction, global cerebral edema, re-bleeding, hydrocephalus) in sedated patients. Regarding the definition of therapeutic consequences, IHTs to transfer the patient to an intervention (e.g., surgery, angiography) were excluded and only IHTs for the purpose of diagnostic procedures (*n* = 91) were considered. The following three categories were defined: direct therapeutic consequences, decision to limit therapy, or no therapeutic consequences. To classify the individual cases, EHRs were reviewed by one author (TLTW) and validated by a second author (MLS).

### 2.4. Definition of Clinical Thresholds and Combined Endpoints for IHT Complications

In addition to the mean change in variables before and after transport, we classified post-IHT complications based on deviations from predefined clinical thresholds (c—critical). As the literature only reports thresholds for IHTs in brain-injured patients in general [5,7], we defined cutoffs for IHT in SAH patients as a consensus among the authors based on clinical expertise. Limits were defined as a decrease in the GCS by more than two points (cGCS), decrease in mean arterial pressure (cMAP min) below 65 mmHg, systolic blood pressure greater than 180 mmHg (cSBP max), heart rate below 50 (cHR min) or above 160 (cHR max), oxygen saturation (cSO_2_) below 92%, pH below 7.2 (cpH), carbon dioxide concentrations below 33 (cCO_2_ min) or above 45 (cCO_2_ max), lactate above 4 mmol/L (cLac), glucose below 80 mg/dL (cGlu min) or above 180 mg/dL when the pre-transport level was below 180 mg/dL (cGlu max), decrease in the Horovitz quotient by more than 50 mmHg (cHorovitz), temperature (cTemp) below 34° Celsius, intracranial pressure (cICP) above 22 mmHg when the pre-transport measure was beneath this value, and cerebral perfusion pressure (cCPP) below 70 mmHg. Additionally, the introduction or placement of new central lines within 24 h of IHT (cCentral lines), the renewal of an EVD within 24 h of IHT (cEVD new), or a previously drained EVD showing no drainage after IHT (cEVD not draining) were also defined as critical events. Combined endpoints for hemodynamic (either cSBP max, cMAP min or cHR min being critically altered), respiratory (either cHorovitz, cCO_2_ min or cCO_2_ max being critically altered), and neurological complications (either cICP or cCPP being critically altered) were defined as composite outcomes. Frequencies of these complications were determined for single and combined endpoints, respectively.

### 2.5. Statistical Analysis

All continuous data were described as mean ± SD. Categorical data were represented using medians with interquartile ranges (IQR). After exploring the data with a Q-Q plot for normal distribution, the Wilcoxon matched-pairs signed-rank test was chosen to compare the paired observations of pre- and post-IHT variables.

As different means might not represent clinically meaningful events, we used the composite outcomes specified above to test potential predictors of complications. Given the wide range of potential predictors and the limited availability of outcomes, we deployed a two-staged approach to (a) achieve a meaningful event to predictor ratio and (b) respect the hierarchical nature of the data. Initially, we screened for statistically significant variables (univariable screening). As the rationale is to avoid prematurely discarding variables (Type II error), we refrained from correcting for multiple tests at this stage. In a second step, multivariate logistic regression corrected with cluster-robust standard errors was used. For univariable screening, co-linearity was analyzed using the Variance Inflation Factor (VIF), and variables with a value above 10 were subsequently excluded (GCS at ICU admission, WFNS, systolic blood pressure variability, MAP variability). The remaining independent variables were age, sex, APACHE II at admission, time since admission, and reason for IHT, as well as the following parameters as recorded immediately before transport: GCS, average MAP, average systolic blood pressure, heart rate, pH, CO_2_, temperature, ICP, CPP, midazolam dosage, propofol dosage, ketamine dosage, sufentil dosage, noradrenaline dosage, lactate level, glucose level, FiO_2_, tidal volume, PEEP, and Horovitz index. The differences between groups with versus without complications were assessed using univariate logistic regression for categorial data and the Mann–Whitney U Test for continuous data, respectively. In the multivariate analysis, multivariate logistic regression was used. Here, 95% confidence intervals and *p*-values were adjusted to reflect the clustering of datapoints (cluster-robust standard errors). To establish clinically useful thresholds for predictors, Youden’s J was calculated. We refrained from modeling clinical outcomes (modified Rankin Scale) due to the mismatch of available endpoints (n = 24) and the high number of necessary covariates. Statistical significance was assumed at *p* < 0.05. Analysis was executed using R software, version 2023.06.1 + 524. The study predominantly harnessed the glmnet, pROC, stats, and mice packages. Missing data, which amounted to 11.8% of the pre/post-IHT variables, were imputed using multiple imputation with predictive mean matching.

## 3. Results

### 3.1. Clinical Characteristics

**Figure 2:**
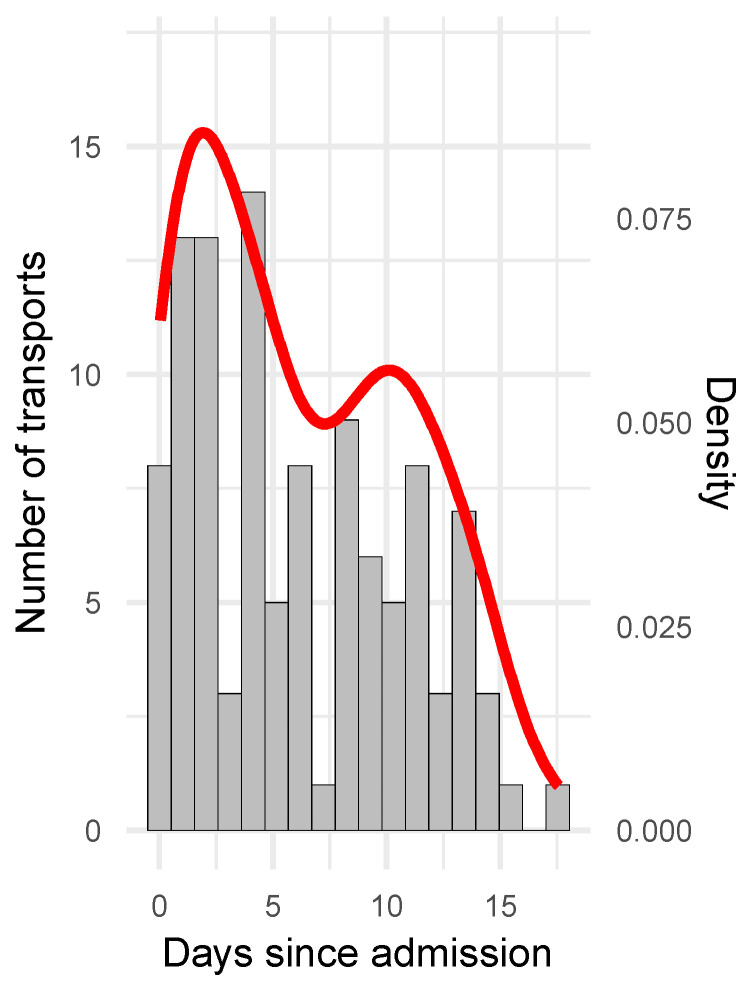
Timing of IHTs. Distribution of IHTs over time is indicated with bars (number of transports per days (grey), left y-axis) and density plot (likelihood of data falling into the respective bin (red), right y-axis). First transport per patient occurred at a mean of 1.5 (+/−2.0) days post admission.

Baseline characteristics of patients and the corresponding IHTs are depicted in Table 1 and Table 2, respectively. Patients included in this cohort had a mean age of 55.7 years (+/−15.3), were predominantly female (72%), critically ill (68% WFNS 5, APACHE II 24 (IQR 19–26)), and showed a remarkably good outcome after NICU treatment (mRS at last possible follow up 1.5 (IQR 0–4.5)). Within 14 days, patients received a mean of 4.3 (SD 1.8) IHTs (Table 2 and Figure 2). Notably, the vast majority of the IHTs performed for diagnostic purposes (n = 91) were scheduled routine transports (post-clamping CTs 16.5%, n = 15; post-interventional CTs 15.4%, n = 14; other routine evaluations 37.4%, n = 34), with emergency IHTs occurring in 30.8% (n = 28) of cases.

A substantial portion of the transports (38.5%, n = 35), resulted in direct therapeutic consequences. Yet, the majority (58.2%, n = 53) resulted in no direct therapeutic consequences. In three cases (3.3%), a decision to withdraw therapy was made (Table 2).

### 3.2. Complications of IHTs

Comparing key parameters of hemodynamic, respiratory, metabolic, and neurological function before and after transport, there were significant changes in the average MAP (pre-versurs post-IHT, 85.7 (+/−9.4) mmHg to 88.9 (+/−11.6) mmHg, *p* < 0.001), average systolic blood pressure (136.3 (+/−14.1) mmHg to 139.4 (+/−15.5) mmHg, *p* = 0.014), CPP (76.9 (+/−14.2) mmHg to 80.6 (+/−15.9) mmHg, *p* = 0.007), propofol dosage (74.1 (+/−120.5) mg/h to 123.1 (+/−151.7) mg/h, *p* < 0.001), and ketamine dosage (196.9 (+/−165.2) mg/h to 229.3 (+/−160.4) mg/h, *p* = 0.035) (Supplementary Figure S1 and Table S1). Additionally, there were significant shifts in oxygen saturation (97.4 (+/−2.1)% to 96.5 (+/−4.4)%, *p* = 0.014), FiO_2_ (41.4 (+/−13.2)% to 46.5 (+/−18.2)%, *p* < 0.001), tidal volume (588.7 (+/−144.8) mL to 616.3 (+/−165.9) mL, *p* = 0.022), Horovitz index (276.7 (+/−123.4) to 266.3 (+/−140.7), *p* = 0.009), glucose (137.2 (+/−46.7) to 129.8 (+/−34.2) mg/dL, *p* = 0.0181), and body temperature (36.9 (+/−0.6) °C to 36.7 (+/−0.8) °C, *p* = 0.002) (Supplementary Figure S1 and Table S1).

As these differences in means might not be clinically meaningful, we also analyzed the frequency of violations of pre-defined critical thresholds relative to the totality of IHTs and per patient (for definition see methods, Section 2.3) (Table 3). Here, most frequent deviations were observed with regards to mechanical ventilation (critical hyperventilation (cCO_2_ min) 38.0%, n = 41, critical decline in oxygenation (cHorovitz) 24.7%, n = 24, combined respiratory endpoint 63.9%, n = 69), and hemodynamics (critical hypertension (cSBP max) 20.6%, n = 22, combined hemodynamic endpoint 31.5%, n = 34). Interestingly, accidental damage or even removal of central lines or EVDs was also quite frequent (new central line within 24 h after IHT (cCentral lines), 7.6%, n = 8; non-draining EVDs (cEVD not draining) 11.5%, n = 12; new EVD within 24 h after IHT (cEVD new) 15.2%, n = 15).

### 3.3. Predictors of IHT Complications

**Figure 3:**
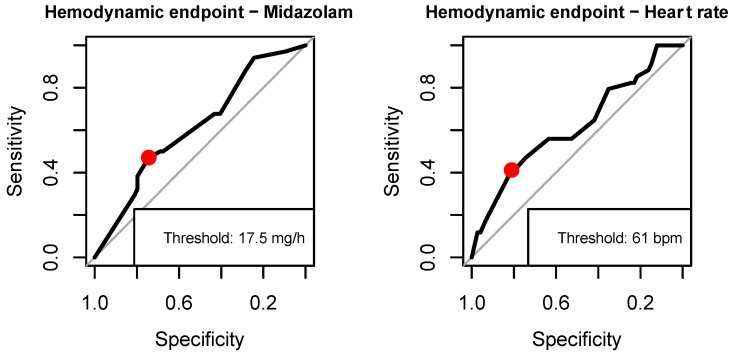
Thresholds for dosage of midazolam and heart rate for hemodynamic complications.

With respect to the composite outcomes, we found age (no IHT complications vs. complications, 52.8 (+/−16.8) vs. 61.8 (+/−12.5) years, *p* = 0.0098), heart rate (76.5 (+/−14.8) vs. 69.9 (+/−13.6) beats per minute, *p* = 0.0478), and dosage of midazolam (30.3 (+/−19.2) vs. 22.1 (+/−18.7) mg/h, *p* = 0.0498) to be predictors of hemodynamic complications, and dosage of noradrenalin (0.5 (+/−0.7) vs. 0.7 (+/−0.8), *p* = 0.0417) to be significantly associated with respiratory complications in the univariate analysis (Table 4). With logistic regression and adjustment for cluster effects, the independent variables heart rate and dosage of midazolam in the hemodynamic model remained significant, but exhibited small effect sizes (heart rate (OR 0.96 (0.92–1.00), *p* = 0.0335), dosage of midazolam (OR 0.97 (0.95–1.00), *p* = 0.0383)) (Table 5) and low-to-moderate predictive performance (area under the curve receiver operating characteristic, AUC-ROC midazolam 0.62, AUC-ROC heart rate 0.62, AUC-ROC multivariate model 0.68). The thresholds for significant predictors indicating the optimal discrimination between complications versus the absence of complications on IHT were a heart rate of 61/minute and a dosage of midazolam of 17.5 mg/h, respectively (Figure 3).

## 4. Discussion

In this cohort study on critically ill SAH patients, we evaluated the timing, nature, and frequency of relevant complications including their respective predictors, and the clinical consequences of IHT. The main findings of this study are: (i) the majority of IHTs follow a routine indication and are performed to allow diagnostic procedures; (ii) around 40% resulted in direct clinical consequences; (iii) clinically meaningful thresholds are violated in 31.5% for hemodynamic endpoints, 63.9% for respiratory endpoints, and 20.4% for neurological endpoints; and (iv) a heart rate below 61/minute and midazolam dosage lower than 17.5 mg/h are predictors of hemodynamic complications.

The literature reveals varying frequencies of emergency indications for IHTs in brain-injured patients, ranging from 17.8% to 42.0% [6,7,8]. Our study aligns with this, recording that 30.8% of IHTs were due to clinical emergencies. Similarly, we found that 84.3% (91/108) of IHTs had diagnostic purposes, while for a comparable population, a frequency of 79.8% is reported [8]. The same study supports our findings regarding timing, showing IHTs are conducted throughout the entire ICU stay. Our data, as well asseveral prior studies, suggest that neither emergency indications for IHT, nor timing are independent risk factors for complications in brain injured patients [5,6,8]. In contrast, another study with neurosurgical patients and a similar definition of clinical emergencies compared to our study (new onset aniscoria, raised ICP) reports IHTs to be associated with a higher rate of complications [7]. Given that most of the poor-grade SAH patients are sedated in the early phase of hospitalization [9], and are thus not eligible for monitoring via clinical examination, the high frequency of routine indications for IHT is largely related to regular transfers to perform cranial imaging [8]. Recent data back this practice by providing evidence for CT angiography (CTA) and CT perfusion (CTP) to help in deciding on the management of potential vasospasm and delayed cerebral ischemia (DCI) [10,11,12].

This practice of routine imaging in SAH, even without strong clinical suspicion, underscores the importance of understanding the risks and potential benefits of IHTs. Martin et al. investigated the risks associated with IHTs for routine CT by day 3 after severe traumatic brain injury (TBI) and found that a therapeutic consequence could be drawn from the available imaging in only 1/31 patients. In contrast, in another neurosurgical cohort, Bender et al. reported therapeutic consequences in over 2/3 of the studied population [7]. Beyond this, data on the therapeutic implications of imaging in SAH are lacking in the literature. While the number will undoubtedly vary depending on the cohort and its demographics, it is notable that, with comparable definitions of the endpoint, roughly 40% of IHTs to cranial imaging, to some extent, resulted in a change in management (e.g., adapting ICU management, surgery, withdrawal of therapy) in our cohort of critically ill SAH patients. Taken together, between 30–60% of IHTs to perform neuroimaging in the neurocritical care population remain without immediate therapeutic consequence. This highlights the need for (a) better patient selection and (b) alternative strategies to monitor patients and cerebral metabolism at the bedside. In this context, establishing additional real-time, whole-brain neuromonitoring with continuous electroencephalography (EEG) or deploying bedside imaging techniques such as transcranial ultrasound or portable CTs/MRIs are promising approaches [13,14].

It is crucial to recognize that, while many parameters were significantly altered after IHT compared to their baseline values, this might not be of clinical significance. Therefore, as in previous studies on IHTs, we also established thresholds and adapted them to the specific pathophysiology of SAH. However, this makes a comparison with pre-existing literature difficult, as thresholds vary among studies and their respective populations [3]. In the general ICU population, a recent meta-analysis estimated the frequency of patient-related complications with an alteration in vital signs as well as procedural complications such as equipment failure to be 26.2% with high heterogeneity among studies [3]. A study conducted with TBI patients observed a composite outcome of critical changes in neurological (ICP, CPP), respiratory (oxygen saturation), and hemodynamic (SBP) parameters in 52% of patients during IHT, but only in 13% upon return to the ICU after IHT [5]. Significant changes in CPP and ICP during CT and during transport back to the ICU were found in SAH patients, but not in the post-processing phase after returning to the ICU [6]. Interestingly, our results also demonstrate a considerable number of complications in this post-IHT phase on the NICU, indicating the higher vulnerability of respiratory and hemodynamic parameters as compared to neurological parameters.

In severe TBI with early IHT, age, higher pre-IHT ICP levels, and a higher dosage of noradrenalin were associated with secondary brain damage in a univariate analysis [5]. Yet, in our data, the dosage of noradrenalin was not a significant predictor for respiratory complications. In another cohort with indwelling EVD, including patients with SAH, high-volume drainage, high baseline ICP levels before transport, and IHT for therapeutic procedures were predictors of ICP crisis in the context of IHT. Interestingly, patients who already had their EVD clamped before IHT did not suffer any complications. In contrast to these trials, our study had a very low frequency of neurological complications overall (1/99 ICP crisis, 15/98 low CPP), potentially reflecting the different time points of data collection (during versus after IHT). Moreover, as substantially increased ICP was only present in one case after IHT, the observed low CPP can be regarded as a surrogate of the hemodynamic complications rather than a consequence of elevated ICP. Other studies have suggested that the process of transporting a patient using elevators or ramps and transferring the patient from the bed onto the gantry or the operating table are associated with spikes in ICP and altered brain metabolism [2,4]. As we focused on the phase upon return to the ICU, short violations of the ICP threshold might have been missed in our study. Yet, it is reasonable to assume that the overall ICP burden (intensity over time), which has been shown to be the relevant biomarker for outcome [15,16], is not substantially altered in cases with normal ICP upon arrival at the ICU after IHT. Despite prior results suggesting a role for hemoglobin as a serum biomarker for predicting neurological complications during IHT [7], we did not observe any interaction with any blood-based parameters, given the overall low event rate of ICP complications.

While the majority of studies in the neurocritical care population focused primarily on IHT-associated secondary brain-damage, we broadened the spectrum of independent variables, also encompassing a range of hemodynamic, respiratory, and metabolic factors. Thereby, we identified lower amounts of sedation (midazolam) and low heart rate as predictors of hemodynamic complications. While low amounts of sedatives favoring hemodynamic instability might seem counterintuitive at first, it seems reasonable to conclude that inadequately low sedation during IHT contributes to agitation and thus may be associated with consecutive hemodynamic complications. Yet, other sedatives, especially propofol, were not identified as a relevant independent variable in our study. Data from the general ICU population with different endpoints (pooling of various patient-related adverse events such as hypotension, oxygen desaturation, and agitation as a combined endpoint) indicated that PEEP > 6 cm H_2_O and treatment modifications before the start of the IHT are risk factors for IHT-associated complications. Notably, both age and sedation were not significantly associated with this endpoint, while heart rate was not investigated as a variable [17].

Even though IHTs are undoubtably hazardous, guidelines from international intensive care consortia have not been updated for the last 20 years [18,19,20]. However, a number of checklists for IHT have been proposed by individual authors recently, including a study specifically addressing neurocritical care patients [21,22,23]. As these checklists mostly focus on equipment-related factors and risk stratification, the competency of the staff involved remains unaddressed. To tackle this issue, safely performing an intrahospital transport was defined as one of seven entrustable professional activities (EPAs) needed for being on-call on a NICU [24].

In addition to the comprehensive analysis beyond just neurological parameters, other strengths of our analysis include the simultaneous reporting of surrogates for risks (frequency of parameter change and clinical complications) and benefits (therapeutic consequences) specifically for SAH patients, which has, to the best of our knowledge, never been described before. Furthermore, we respected the clustering of data due to several datapoints per patient and are able to provide concrete thresholds for risk factors associated with IHT-associated complications facilitating both further research and clinical decision making. Our study is limited by the small sample size and the shortcomings inherent to the study design of a monocentric, retrospective study. Specifically, local protocols, equipment, and expertise of clinical personal limit external validity. In addition, the outcomes and computed risk factors greatly depend on the thresholds set for critical events, which—in the absence of an adequate number of published studies for this cohort—we were not able to create a fully literature-informed strategy. Furthermore, events during transport as well as near misses and non-patient related factors such as equipment failure without secondary effects on the patient’s parameters were not recorded. While such procedural data are important for quality management, previous data show that team-, environment-, equipment-, organization- and skill-related errors are rather rare [25].

## 5. Conclusions

In summary, this study provides data to support clinical decision making by portraying the risks and benefits of IHTs in SAH patients. As around 40% of IHTs have an immediate clinical consequence, using age, level of sedation, and heart rate as risk factors for hemodynamic complications may be useful to optimize patient selection. Further validation with prospective multicenter studies is warranted.

## Figures and Tables

**Table 1:** Clinical characteristics and outcomes of SAH patients.

Age [years], mean (SD)		55.7 (15.3)
Female sex, *n* (%)		18/25 (72.0)
APACHE II, median (IQR)		24 (19–26)
WFNS scale, *n* (%)	1	-
	2	2/25 (8.0)
	3	3/25 (12.0)
	4	3/25 (12.0)
	5	17/25 (68.0)
Modified Fisher scale, *n* (%)	0	-
	1	-
	2	1/25 (4.0)
	3	10/25 (40.0)
	4	24/25 (56.0)
Aneurysm location, *n* (%)	ACA	2/25 (8.0)
	MCA	5/25 (20.0)
	PCA	1/25 (4.0)
	CommA	4/25 (16.0)
	CommP	2/25 (8.0)
	BA	6/25 (24.0)
	ICA	3/25 (12.0)
	None detected	2/25 (8.0)
Mode of treatment, *n* (%)	Endovascular	14/25 (56.0)
	Surgical	8/25 (32.0)
	None	3/25 (12.0)
ICU length of stay [days], mean (SD)		29.2 (17.8)
DCI, n (%)		7/25 (28.0)
Death during ICU treatment, *n* (%)		5/25 (20.0)
mRS at discharge from ICU, median (IQR)		2.5 (0–5)

SD—standard deviation; IQR—interquartile range; APACHE II–Acute Physiology and Chronic Health Evaluation II; WFNS—World Federation of Neurosurgical Societies; ACA—anterior cerebral artery; MCA—middle cerebral artery; PCA—posterior cerebral artery; CommA—anterior communicating artery; CommP—posterior communicating artery; BA—basilar artery; ICA—internal communicating artery; ICU—intensive care unit; DCI—delayed cerebral ischemia; mRS—modified Rankin Scale.

**Table 2:** Indications and clinical consequences of IHT for diagnostic purposes.

Transports Per Patient, Mean (SD)		4.3 (1.8)
Reason for transport, *n* (%)	Clinical emergency	28/91 (30.8)
	Scheduled control after intervention	14/91 (15.4)
	Post-clamping CT	15/91 (16.5)
	Other routine IHTs	34/91(37.4)
Therapeutic consequences, *n* (%)	Direct therapeutic consequences	35/91 (38.5)
	Decision to limit therapy	3/91 (3.3)
	No immediate therapeutic consequence	53/91 (58.2)

SD—standard deviation; CT—computed tomography; IHT—intra-hospital transport.

**Table 3:** Frequency of IHT complications.

Event	Frequency (% of IHTs), n (%)	Per Patient, Mean (SD)
cGCS	0/93 (0)	0 (0)
cMAP min	11/108 (10.2)	0.4 (0.7)
cSBP max	22/107 (20.6)	0.9 (1.0)
cHR min	5/108 (4.6)	0.2 (0.6)
cHR max	0/108 (0)	0 (0)
cSO_2_	7/108 (6.5)	0.3 (0.6)
cpH	6/108 (5.6)	0.2 (0.5)
cCO_2_ min	41/108 (38.0)	1.6 (1.3)
cCO_2_ max	20/108 (18.5)	0.8 (1.2)
cLac	2/108 (1.9)	0.1 (0.3)
cGlu min	8/108 (7.4)	0.3 (0.7)
cGlu max	14/108 (13.0)	0.6 (0.9)
cHorovitz	24/97 (24.7)	1.2 (1.2)
cTemp	0/10 (0)	0 (0)
cICP	1/99 (1.0)	0.1 (0.3)
cCPP	15/98 (15.3)	0.8 (1.2)
cCentral lines	8/106 (7.6)	0.3 (0.6)
cEVD new	15/99 (15.2)	0.8 (0.9)
cEVD not draining	12/104 (11.5)	0.5 (0.8)
Combined hemodynamic endpoint	34/108 (31.5)	1.4 (1.2)
Combined respiratory endpoint	69/108 (63.9)	2.8 (1.5)
Combined neurological endpoint	22/108 (20.4)	0.9 (1.2)

GCS—Glasgow Coma Scale; MAP—mean arterial pressure; SBP—systolic blood pressure; HR—heart rate; SO_2_—oxygen saturation; CO_2_—carbon dioxide; Lac—lactate; Glu—glucose; Temp—temperature; ICP—intracranial pressure; CPP—cerebral perfusion pressure; EVD—external ventricular drain; SD—standard deviation.

**Table 4:** Univariate analysis of predictors of the composite outcomes for hemodynamic, respiratory, and neurological complications.

	Endpoint	No IHT Complication, Mean (SD)	IHT Complication, Mean (SD)	*p*-Value
Age	Hemodynamic	52.8 (16.8)	61.8 (12.5)	**0.0098**
	Respiratory	55.5 (16.4)	55.7 (16)	0.9744
	Neurological	55.2 (15.6)	57.1 (18.1)	0.3148
APACHE II	Hemodynamic	22.3 (6.2)	19.7 (7.1)	0.0874
	Respiratory	21.0 (7.5)	21.7 (6.0)	0.9974
	Neurological	21.8 (6.1)	20.4 (8.2)	0.9420
Time since admission	Hemodynamic	155.3 (109.4)	136 (119.2)	0.4196
	Respiratory	165.7 (109.0)	139.9 (114.0)	0.1492
	Neurological	147.6 (115.6)	155.6 (101.0)	0.5341
GCS before IHT	Hemodynamic	4.5 (3.9)	4.8 (4.3)	0.4800
	Respiratory	5.2 (4.7)	4.2 (3.6)	0.1482
	Neurological	4.4 (3.9)	5.2 (4.7)	0.5041
MAP	Hemodynamic	86.5 (8.8)	84 (10.4)	0.2635
	Respiratory	87.2 (10.1)	84.9 (8.9)	0.2712
	Neurological	86.6 (9.2)	82.4 (9.6)	0.0616
Heart rate	Hemodynamic	76.5 (14.8)	69.9 (13.6)	**0.0478**
	Respiratory	74.1 (13.7)	74.6 (15.3)	0.9233
	Neurological	75.1 (15.1)	71.5 (12.9)	0.3520
pH	Hemodynamic	7.4 (0.1)	7.4 (0.1)	0.3075
	Respiratory	7.4 (0.1)	7.4 (0.1)	0.7366
	Neurological	7.4 (0.1)	7.4 (0.1)	0.6883
CO_2_	Hemodynamic	38.2 (7.8)	38.1 (6.8)	0.8041
	Respiratory	39.0 (5.6)	37.7 (8.3)	0.1386
	Neurological	38.3 (7.6)	37.5 (7.0)	0.4828
Body temperature	Hemodynamic	36.9 (0.6)	36.8 (0.6)	0.6655
	Respiratory	37.0 (0.6)	36.8 (0.6)	0.2524
	Neurological	36.9 (0.6)	36.8 (0.8)	0.5940
ICP	Hemodynamic	11.0 (5.6)	10.9 (6.4)	0.8882
	Respiratory	11.6 (5.9)	10.6 (5.8)	0.2871
	Neurological	11.1 (5.4)	10.4 (7.2)	0.3065
CPP	Hemodynamic	77.7 (13.1)	75.1 (16.4)	0.5832
	Respiratory	73.6 (14.7)	78.8 (13.7)	0.0780
	Neurological	76.9 (13.2)	77 (18.1)	0.9236
Midazolam	Hemodynamic	30.3 (19.2)	22.1 (18.7)	**0.0498**
	Respiratory	27.3 (18.7)	28 (19.8)	0.9380
	Neurological	28.4 (17.9)	25.2 (24.5)	0.5802
Propofol	Hemodynamic	60.1 (112.9)	104.4 (132.2)	0.0505
	Respiratory	64.1 (120.3)	79.7 (121.1)	0.4228
	Neurological	73.3 (118.7)	77.3 (129.8)	0.9371
Sufentanil	Hemodynamic	27.9 (18.7)	26.4 (17.6)	0.8559
	Respiratory	28.8 (19.0)	26.7 (17.9)	0.5302
	Neurological	27.3 (16.9)	28.1 (23.2)	0.8462
Ketamin	Hemodynamic	209.1 (162.6)	170.3 (170.2)	0.2882
	Respiratory	179.2 (147.7)	206.8 (174.6)	0.3411
	Neurological	190.3 (159.7)	222.3 (187.1)	0.4368
Noradrenalin	Hemodynamic	0.7 (0.8)	0.5 (0.6)	0.2210
	Respiratory	0.5 (0.7)	0.7 (0.8)	**0.0417**
	Neurological	0.5 (0.7)	1.0 (1.1)	0.1182
SBP	Hemodynamic	135.8 (13.9)	137.3 (14.7)	0.4913
	Respiratory	139.8 (15.4)	134.3 (13.0)	0.0582
	Neurological	136.5 (14.1)	135.2 (14.6)	0.5466
Lactate	Hemodynamic	1.2 (1.8)	1.6 (2.8)	0.4958
	Respiratory	1.3 (2.4)	1.4 (2.0)	0.5331
	Neurological	1.2 (1.8)	1.8 (3.2)	0.2061
Glucose	Hemodynamic	138.1 (53.3)	135.3 (28.2)	0.6058
	Respiratory	134 (36.2)	139.1 (51.9)	0.6451
	Neurological	136.7 (36.9)	139.5 (75.0)	0.3480
FiO_2_	Hemodynamic	40.2 (12.3)	43.8 (14.8)	0.2924
	Respiratory	41.9 (15.2)	41.0 (12.0)	0.8981
	Neurological	42.5 (13.6)	36.9 (10.4)	0.1030
Tidal volume	Hemodynamic	593.6 (146.9)	578.3 (141.8)	0.6407
	Respiratory	598.3 (138.6)	583.3 (149)	0.4794
	Neurological	586.8 (143)	596.4 (155.2)	0.9240
PEEP	Hemodynamic	7.4 (2.7)	7.6 (3.2)	0.9354
	Respiratory	7.4 (3.0)	7.5 (2.8)	0.5656
	Neurological	7.5 (2.8)	7.5 (3.1)	0.8609
Horovitz index	Hemodynamic	285.4 (124.6)	257.8 (120.4)	0.4312
	Respiratory	280.9 (129.5)	274.4 (120.8)	0.9974
	Neurological	281 (118.2)	260.2 (144)	0.4876
		Endpoint	OR (95% CI)	*p*-value
Sex (Ref. female sex)		Hemodynamic	1.4 (0.5–3.7)	0.4728
		Respiratory	0.9 (0.4–2.7)	0.8724
		Neurological	1.0 (0.3–3.2)	1
Resason for IHT	Other routine IHT (Ref.)			
	Scheduled after intervention	Hemodynamic	1.5 (0.4–5.0)	0.5377
		Respiratory	1.8 (0.5–6.5)	0.4023
		Neurological	0.3 (0–2.2)	0.2104
	Post-clamping CT	Hemodynamic	0.7 (0.2–2.3)	0.6021
		Respiratory	0.7 (0.2–1.9)	0.4571
		Neurological	1.2 (0.3–3.7)	0.8475
	Clinical emergency	Hemodynamic	1.6 (0.6–4.5)	0.3800
		Respiratory	1.2 (0.4–3.5)	0.6923
		Neurological	1.3 (0.4–4.0)	0.7056

APACHE II—Acute Physiology and Chronic Health Evaluation II; GCS—Glasgow Coma Scale; MAP—mean arterial pressure; CO_2_—carbon dioxide; ICP—intracranial pressure; CPP—cerebral perfusion pressure; SBP—systolic blood pressure; FiO_2_—fraction of inspired oxygen; PEEP—positive end expiratory pressure; IHT—intra-hospital transport; OR—odds ratio; CT—computed tomography.

**Table 5:** Logistic regression with cluster-robust standard errors (CRSE) for hemodynamic and respiratory complications.

		Odds Ratio	Adjusted 95% CI	*p*-Value
Hemodynamic	Age	1.04	1.00–1.08	0.0591
	Heart rate	0.96	0.92–1.00	**0.0335**
	Midazolam	0.97	0.95–1.00	**0.0383**
Respiratory	Noradrenalin	1.5	0.8–2.7	0.1694

CI—Confidence interval.

## Data Availability

Data are contained within the article or Supplementary Materials. The data presented in this study are available in Supplementary Table S2.

## Supplementary Materials

The following supporting information can be downloaded at: https://www.mdpi.com/article/10.3390/jcm12247666/s1, Figure S1 and Table S1: Parameters pre and post IHT. Table S2: raw data.
