# Supplementary material for: Intrahospital Transport of Critically Ill Patients with Subarachnoid Hemorrhage—Frequency, Timing, Complications, and Clinical Consequences
Source: J Clin Med. 2023 Dec 13;12(24):7666. doi: 10.3390/jcm12247666 (PMC10743394; doi:10.3390/jcm12247666)
Supplement: Supplementary file 1 [file jcm-12-07666-s001.zip › 09_23_SAH_transport_JCM_Supplemental 1.docx]

**Supplemental Figure 1.** Boxplots of changes for hemodynamic, respiratory, neurological and metabolic parameters after IHT

**Supplemental Table 1**. Mean and standard deviations for for hemodynamic, respiratory, neurological and metabolic parameters after IHT

| **Variables** | **Mean_Pre** | **SD_Pre** | **Mean_Post** | **SD_Post** | **p_value** |
| --- | --- | --- | --- | --- | --- |
| **GCS** | 4.6 | 4.0 | 3.8 | 3.0 | 1.0000 |
| **MAP average** | 85.7 | 9.4 | 88.9 | 11.6 | <0.001 |
| **MAP variability** | 7.0 | 5.6 | 7.6 | 5.6 | 0.3651 |
| **Heart rate** | 74.4 | 14.7 | 74.2 | 16.1 | 0.8726 |
| **Oxygen saturation** | 97.4 | 2.1 | 96.5 | 4.4 | 0.0137 |
| **pH** | 7.4 | 0.1 | 7.4 | 0.1 | 0.7713 |
| **CO_2_** | 38.2 | 7.4 | 37.1 | 9.0 | 0.1191 |
| **Body temperature** | 36.9 | 0.6 | 36.7 | 0.8 | 0.0022 |
| **ICP** | 11.0 | 5.8 | 10.2 | 7.7 | 0.1162 |
| **CPP** | 76.9 | 14.2 | 80.6 | 15.9 | 0.0072 |
| **Midazolam** | 27.7 | 19.3 | 30.9 | 20.0 | 0.0860 |
| **Propofol** | 74.1 | 120.5 | 123.1 | 151.7 | <0.001 |
| **Sufentanil** | 27.5 | 18.3 | 28.1 | 17.3 | 0.4967 |
| **Ketamine** | 196.9 | 165.2 | 229.3 | 160.4 | 0.0346 |
| **Noradrenalin** | 0.6 | 0.8 | 0.6 | 0.8 | 0.5760 |
| **SBP average** | 136.3 | 14.1 | 139.4 | 15.5 | 0.0142 |
| **SBP variability** | 11.2 | 9.2 | 12.8 | 9.8 | 0.1399 |
| **Lactate** | 1.3 | 2.2 | 1.3 | 2.3 | 0.2386 |
| **Glucose** | 137.2 | 46.7 | 129.8 | 34.3 | 0.0181 |
| **Hemoglobin** | 10.4 | 2.2 | 10.2 | 2.1 | 0.0667 |
| **FiO_2_** | 41.4 | 13.2 | 46.5 | 18.2 | <0.001 |
| **Tidal volume** | 588.7 | 144.8 | 616.3 | 165.9 | 0.0223 |
| **PEEP** | 7.5 | 2.9 | 7.6 | 3.0 | 0.5739 |
| **Horovitz_index** | 276.7 | 123.4 | 266.3 | 140.7 | 0.0091 |

GCS – Glasgow Coma Scale, MAP – mean arterial pressure, SBP – systolic blood pressure, CO_2_ – carbon dioxide, Lac – lactate, Glu – glucose, Temp – temperature, ICP – intracranial pressure, CPP – cerebral perfusion pressure, EVD – external ventricular drain
